# Early Changes in Cardiac Macrophage Subsets in Heart Failure with Preserved Ejection Fraction

**DOI:** 10.3390/ijms262010196

**Published:** 2025-10-20

**Authors:** Danae Gutiérrez, Karina Cordero, Ruth Sepúlveda, Camilo Venegas, Diego Altamirano, Camila Candia, Gigliola Ramírez, Patricio Araos, Cristian A. Amador, Marcela A. Hermoso, Luigi Gabrielli, Jorge E. Jalil, María Paz Ocaranza

**Affiliations:** 1 Division of Cardiovascular Diseases, School of Medicine, Pontificia Universidad Católica de Chile, Diagonal Paraguay 362, Piso 7, Santiago 7820436, Chile; danae.gutierrez@uc.cl (D.G.); rangelicasepulveda@gmail.com (R.S.); cvenegao@uc.cl (C.V.); daltamiran@uc.cl (D.A.); camila.candia@uc.cl (C.C.); gigliolaramirez48@gmail.com (G.R.); lgabriel@uc.cl (L.G.); 2 Institute of Biomedical Sciences, Universidad Autonoma de Chile, Santiago 8581151, Chile; patricio.araos@uautonoma.cl; 3 Faculty of Sciences, Universidad San Sebastian, Santiago 8420524, Chile; cristian.amador@uss.cl; 4 Laboratory of Innate Immunity, Program of Immunology, Faculty of Medicine, Institute of Biomedical Sciences, Universidad de Chile, Santiago 7800003, Chile; mhermoso@med.uchile.cl; 5 Advanced Center for Chronic Diseases (ACCDiS), Pontificia Universidad Católica de Chile, Santiago 7820436, Chile; 6 Center of New Drugs for Hypertension and Heart Failure (CENDHY), Division of Cardiovascular Diseases, Pontificia Universidad Católica de Chile, Santiago 7820436, Chile

**Keywords:** heart failure, diabetes, hypertension, cardiometabolic syndrome, macrophages, cytokines, cardiac inflammation

## Abstract

Heart failure with preserved ejection fraction (HFpEF) is a complex syndrome characterized by left ventricular diastolic dysfunction, exercise intolerance, low-grade chronic inflammation, and comorbidities such as hypertension, obesity, and glucose intolerance. Myocardial infiltration by activated macrophages has been proposed as a mechanism linking low-grade inflammation to increased diastolic LV stiffness in HFpEF. Changes in the relative abundance of cardiac macrophage populations may precede and promote the development of HFpEF in the aged heart. This study aimed to characterize the cardiac macrophage subsets that predominate during progression from experimental preclinical to established HFpEF. To generate the model, wild-type male C57BL/6N mice were randomized to control chow or a combination of high-fat diet plus L-NAME in drinking water for 5 weeks (asymptomatic pre-HFpEF) or 15 weeks (established HFpEF). At the end of each period, we measured body weight, running distance, metabolic biomarkers, systolic and diastolic blood pressure, myocardial function and morphology, cardiac remodeling by hypertrophic markers, morphometric analyses, fibrosis, cytokines TNF-α and IL-10, cardiac macrophage phenotype profiles (CCR2^+^ and CCR2^−^), and AMP-Activated Protein Kinase (AMPK)activity.Significant changes in myocardial macrophage populations were observed at 5 weeks (pre-HFpEF), specifically a decrease in resident reparative CCR2^−^MHCII^−^ and increase in proinflammatory CCR2^+^MHCII^+^ macrophages. These early changes were associated with higher circulating TNF-α, decreased myocardial AMPK activation, and more severe myocardial fibrosis. At 15 weeks (established HFpEF), proinflammatory CCR2^+^MHCII^+^ macrophage levels remained elevated in the myocardium; whereas the initial number of resident reparative CCR2^−^MHCII^-^ levels was reduced, it subsequently returned to baseline. In this model of HFpEF induced by a high-fat diet and L-NAME, which produced obesity, glucose intolerance, and hypertension, myocardial resident reparative CCR2^−^MHCII^−^ macrophages decreased and proinflammatory CCR2^+^MHCII^+^ macrophages increased during preclinical stages. These early changes in cardiac macrophage profile were associated with low-grade inflammation and myocardial remodeling and preceded the onset of HFpEF.

## 1. Introduction

Heart failure with preserved ejection fraction (HFpEF) is a clinical syndrome characterized by left ventricular EF (LVEF) ≥ 50% and diastolic dysfunction (DD, impaired LV relaxation/filling). HFpEF is associated with poor quality of life, physical activity limitations, substantial health resource utilization, and premature mortality. HFpEF accounts for >50% of HF cases and is, or will soon become, the dominant HF phenotype, making effective treatment a substantial unmet need in cardiology [1]. Large outcome trials and registries indicate that HFpEF patients tend to be older (≥70 years), are predominantly female, and typically have multiple comorbidities such as overweight/obesity (body mass index > 30 kg/m^2^; 84%), type 2 diabetes mellitus (20–45%), hypertension (HT; 60–80%), renal failure, and sleep apnea. HFpEF is associated with inactivity, atrial fibrillation, echocardiographic abnormalities, elevated B-type natriuretic peptide (BNP ≥ 35 pg/mL or NT-proBNP ≥ 125 pg/mL) [2], and low-grade chronic systemic inflammation [2]. Multimorbidity is common. Most deaths in HFpEF are cardiovascular (CV), but the proportion of non-CV deaths is higher than in HFrEF [3]. Low-grade chronic systemic inflammation has been proposed as a driver of progression from preclinical to symptomatic HFpEF [4]. One mechanism linking low-grade chronic systemic inflammation to increased diastolic LV stiffness is myocardial infiltration by activated macrophages [5].

The normal heart is seeded with resident macrophages that are minimally inflammatory and promote tissue repair. Macrophages are the most abundant immune cells in the heart [6]. These cells maintain tissue homeostasis in healthy myocardium and change in abundance and phenotype in response to tissue injury or disease [6]. Cardiac macrophages are far more transcriptionally heterogeneous; the presence or absence of C-C chemokine receptor type 2 (CCR2) is a robust marker of macrophage origin and phenotype. CCR2 expression distinguishes monocyte-derived cardiac macrophages from those of embryonic origin [7]. The adult heart contains two resident macrophage populations: CCR2^−^MHCII^−^ and CCR2^−^MHCII^+^. CCR2^−^MHCII^+^ macrophages are maintained partly through local proliferation (≈70%) and partly by monocyte replacement (≈30%), whereas CCR2^+^MHCII^+^ (CCR2^+^) macrophages are constantly replaced by monocytes [7]. Resident cardiac macrophages are minimally inflammatory and promote angiogenesis and tissue repair. However, cardiac injury and aging stimulate infiltration of monocyte-derived macrophages that are proinflammatory, promote tissue injury and resident cell death and replacement [7]. The injured adult heart selectively recruits monocytes and CCR2^+^MHCII^+^ monocyte-derived macrophages [8] to prevent/repair myocardial damage. Heart failure due to pressure overload is characterized by T-cell activation, activation that depends on antigenic presentation. The requirement for specific antigen recognition implies an essential pathogenic role for macrophages and other stem cells, although their specific role in the development of pressure overload HF is unclear. Studies have characterized cardiac macrophage populations with differing functions, including tissue-resident, embryonic-derived and infiltrating monocyte-derived macrophages [9].

There is no clinical evidence to date defining the role of macrophages in HFpEF progression, but changes in the relative abundance of these populations may precede and promote HFpEF development in the aged heart. The aim of this study was to determine the role of CCR2 macrophage phenotype predominance in the progression of experimental HFpEF induced by high-fat diet and L-NAME, and its impact on cardiac remodeling and dysfunction.

## 2. Results

### 2.1. HFD+L-NAME Mouse Model Reproduces Major Clinical Features of HFpEF

**Figure 1:**
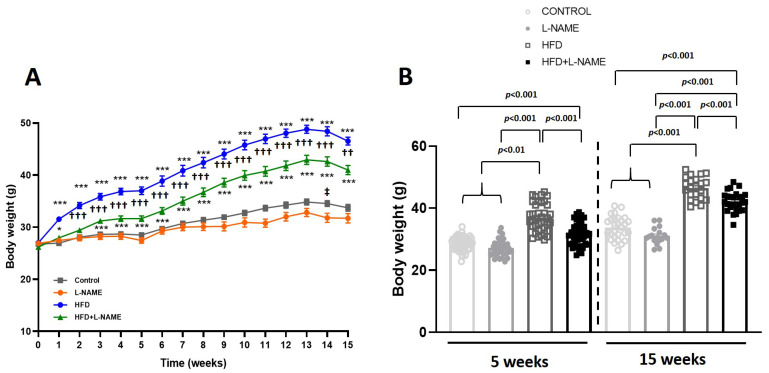
Body weight phenotypes from baseline to 15 weeks of dietary regimens. C57BL/6N mice (12 weeks old) received chow (control, *n* = 8), chow plus L-NAME in drinking water (0.65 g L^−1^, *n* = 8), high-fat diet (HFD, *n* = 12), or HFD+L-NAME (*n* = 12). Results are presented as mean ± s.e.m. One-way analysis of variance (ANOVA) followed by Sidak’s multiple-comparisons test was performed. Numbers above square brackets show significant *p*-values. Symbols: * *p* < 0.05 and *** *p* < 0.001 vs. Control; ^††^ *p* < 0.01 and ^†††^ *p* < 0.001 vs. HFD; ^‡^ *p* < 0.05 vs. L-NAME, after a significant one-way analysis of variance. (**A**). Weight curve of the experimental groups during the treatment (F = 68.4, *p* < 0.001). (**B**). Body weight of mice after the treatments at 5 (F = 56.6, *p* < 0.001) and 15 weeks (F = 89.7, *p* < 0.001).

HFpEF patients commonly exhibit hypertension, obesity, and metabolic dysfunction. We used a two-hit HFpEF model (HFD+L-NAME) [10]. In parallel, four groups of mice received HFD+L-NAME, HFD alone, L-NAME alone, or chow (Figure 1A). As expected in this preclinical model, the weight curve showed that HFD alone produced a greater body weight than HFD+L-NAME (Figure 1A), whereas L-NAME alone did not change body weight vs. vehicle (Figure 1A).

Body weight differed significantly between HFD and HFD+L-NAME groups at week 5 (*p* < 0.001; Figure 1B) and 15 (*p* < 0.001; Figure 1B).

**Figure 2:**
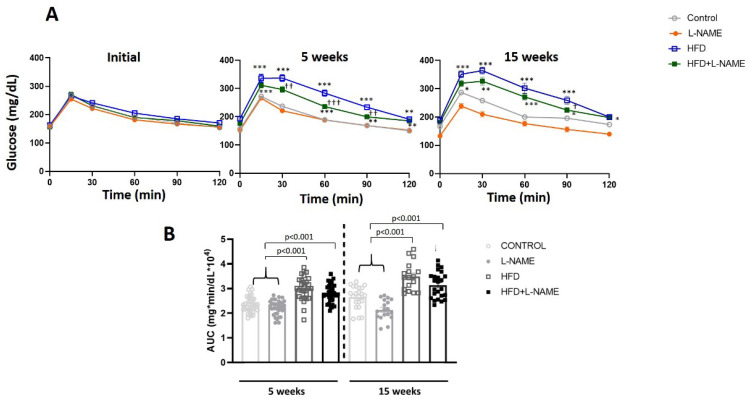
Glucose tolerance after 5 and 15 weeks of different dietary regimens. Groups as in Figure 1. Results are presented as mean ± s.e.m. One-way analysis of variance (ANOVA) followed by Sidak’s multiple-comparisons test was performed. Numbers above square brackets show significant *p*-values. Symbols: * *p* < 0.05; ** *p* < 0.01 and *** *p* < 0.001 vs. Control; ^†^ *p* < 0.05; ^††^ *p* < 0.01 and ^†††^ *p* < 0.001 vs. HFD, after a significant one-way analysis of variance. (**A**) Intraperitoneal glucose tolerance test (ipGTT) at initial, 5 (F = 43.5, *p* < 0.001) and 15 (F = 23.2, *p* < 0.001) weeks after different dietary regimens. (**B**) Area under the curve (mg × min/dl × 10^4^) of the ipGTT experiment at 5 (F = 34.1, *p* < 0.001) and 15 weeks (F = 25.7, *p* < 0.001).

**Figure 3:**
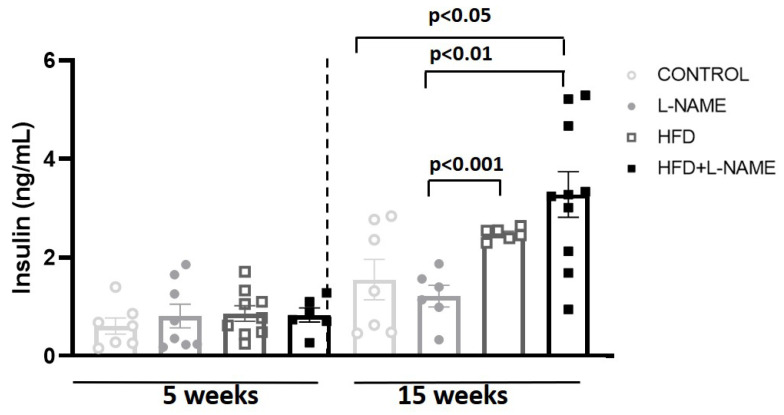
Plasma insulin after 5 and 15 weeks of dietary regimens. Groups as in Figure 1. Results are presented as mean ± s.e.m. One-way analysis of variance (ANOVA) followed by Sidak’s multiple-comparisons test was performed. Numbers above square brackets show significant *p*-values. Plasma insulin levels at 5 (F = 0.374, *p* < 0.78) and 15 weeks (F = 6.2, *p* < 0.004).

Glucose intolerance (ipGTT), measured at baseline, 5, and 15 weeks by intraperitoneal glucose tolerance testing (Figure 2A), increased significantly in the HFD and HFD+L-NAME vs. control and L-NAME groups. The area under the curve for ipGTT (Figure 2B) was also significantly greater in the HFD (*p* ˂ 0.001) and HFD+L-NAME (*p* ˂ 0.001) groups at 5 and 15 weeks. Circulating insulin levels (Figure 3) were significantly higher at 15 weeks in the HFD+L-NAME compared to control (*p* ˂ 0.02) and L-NAME (*p* ˂ 0.006) groups. Finally, insulin levels increased significantly in the HFD (*p* ˂ 0.001, Figure 3) and HFD+L-NAME (*p* ˂ 0.02, Figure 3) groups between weeks 5 and 15.

Four blood lipids, (triglyceride (TG), low-density lipoprotein cholesterol (LDL), high-density lipoprotein cholesterol (HDL), and total cholesterol (CHO)) in mice were evaluated (Table 1). Compared with control and L-NAME mice, TG, LDL, and CHO increased and HDL decreased in the HFD and HFD+L-NAME feeding group, all with statistical significance.

**Figure 4:**
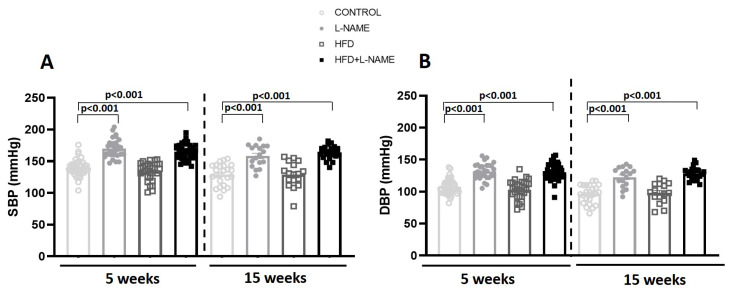
Blood pressure after 5 and 15 weeks of dietary regimens. Groups as in Figure 1. Blood pressure was determined in each group using the indirect tail-cuff method, with a CODA instrument. Results are presented as mean ± s.e.m. One-way analysis of variance (ANOVA) followed by Sidak’s multiple-comparisons test was performed. Numbers above square brackets show significant *p*-values. (**A**). Systolic blood pressure (SBP) at 5 (F = 25.5, *p* < 0.001) and 15 weeks (F = 32.5, *p* < 0.001). (**B**). Diastolic blood pressure (DBP) at 5 (F = 16.5, *p* < 0.001) and 15 weeks (F = 31.3, *p* < 0.001).

**Figure 5:**
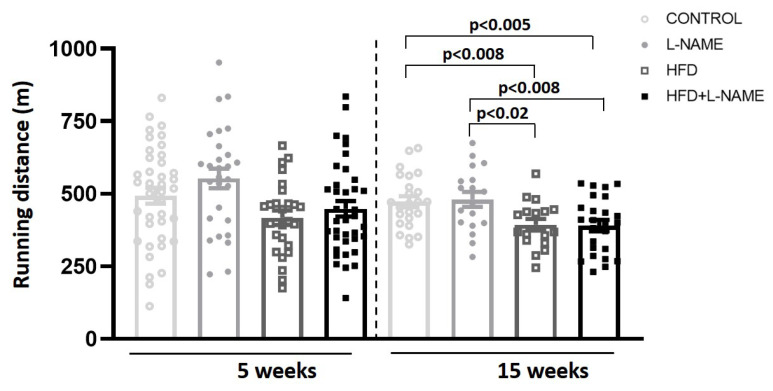
Exercise exhaustion test after 5 and 15 weeks of dietary regimens. Groups as in Figure 1. Results are presented as mean ± s.e.m. One-way analysis of variance (ANOVA) followed by Sidak’s multiple-comparisons test was performed. Numbers above square brackets show significant *p*-values. Running distance at 5 (F = 0.62, *p* < 0.4 and 15 weeks (F = 5.5, *p* < 0.003).

L-NAME and HFD+L-NAME increased both systolic and diastolic blood pressure at 5 and 15 weeks vs. control (*p* < 0.001; Figure 4). Excess weight negatively affected exercise performance at 15 weeks in HFD+L-NAME, with reduced running distance vs. other cohorts (Figure 5).

Longitudinal echocardiography showed preserved LVEF in all groups (Table 2), with significant morphometric changes and diastolic dysfunction at 15 vs. 5 weeks, especially left atrial enlargement in HFD and HFD+L-NAME (Table 2). Increased E/A and E/e′ at 15 weeks indicated more severe diastolic dysfunction. LV hypertrophy, by echo-derived LV mass and LV mass/TL, increased significantly in HFD and HFD+L-NAME at 15 weeks.

**Figure 6:**
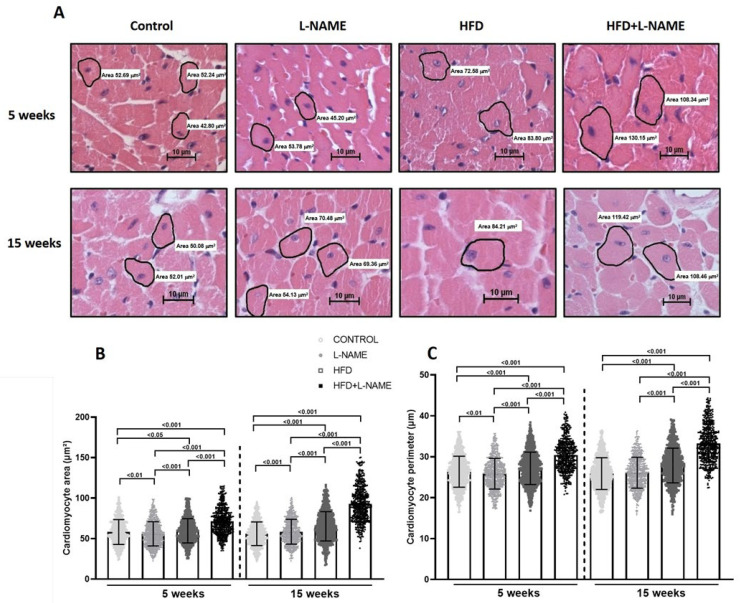
Cardiomyocyte area and perimeter after 5 and 15 weeks of dietary regimens. Groups as in Figure 1. Results are presented as mean ± s.e.m. One-way analysis of variance (ANOVA) followed by Sidak’s multiple-comparisons test was performed. Numbers above square brackets show significant *p*-values. (**A**) Microphotographs of cross-sectional cardiac slices stained with hematoxylin and eosin (400X magnification). Scale bar, 10 µm. (**B**) Cardiomyocyte area at 5 (F = 438.04, *p* < 0.0001) and 15 (F = 268.18, *p* < 0.0001) weeks. (**C**). Cardiomyocyte perimeter in cardiac slices at 5 (F = 219.91, *p* < 0.0001) and 15 weeks (F = 295.83, *p* < 0.0001).

**Figure 7:**
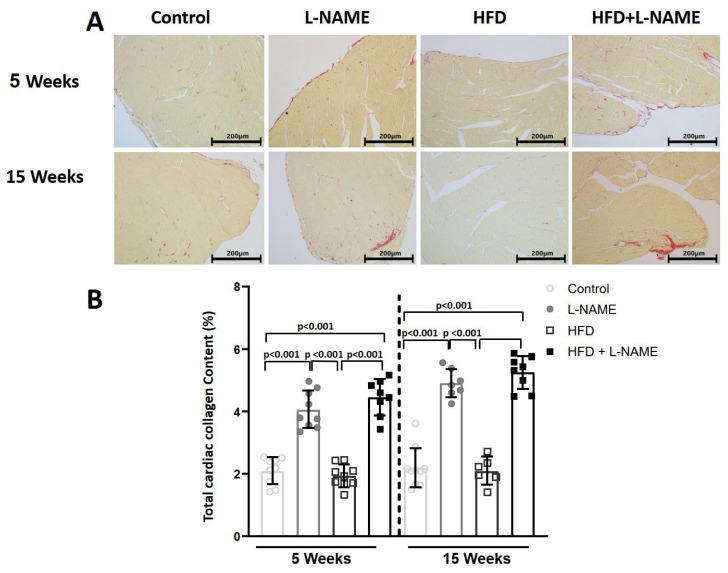
LV fibrosis of mice after 5 and 15 weeks of dietary regimens. Groups as in Figure 1. Results are presented as mean ± s.e.m. One-way analysis of variance (ANOVA) followed by Sidak’s multiple-comparisons test were performed. Numbers above square brackets show significant *p*-values. (**A**). Interstitial collagen volumetric fraction of left ventricle tissue sections stained with Picrosirius red (200X magnification, scale bar = 200 μm) of mice treated with chow diet, L-NAME, HFD and HFD+L-NAME for 5 or 15 weeks. (**B**). Percentage of total LV collagen content of mice treated with chow diet, L-NAME, HFD and HFD+L-NAME for 5 (F = 9.42, *p* < 0.0001) and 15 weeks (F = 9.68, *p* < 0.0001).

Cardiomyocyte area and perimeter as measures of disease-related hypertrophy were higher in HFD plus L-NAME than control, L-NAME and HFD alone (Figure 6A,B) at 5 and 15 weeks. Cardiac fibrosis (Picrosirius Red; Figure 7A) increased significantly in ventricular wall in L-NAME and HFD+L-NAME groups at 5 (both *p* < 0.001) and 15 weeks (both *p* < 0.001) vs. control (Figure 7B).

### 2.2. Progression to Heart Failure with Preserved Ejection Fraction (HFpEF) Is Marked by Early Decrease in Resident Protective CCR2^−^MHCII^−^ Macrophages and Increase in Proinflammatory CCR2^+^MHCII^+^ Macrophages

**Figure 8:**
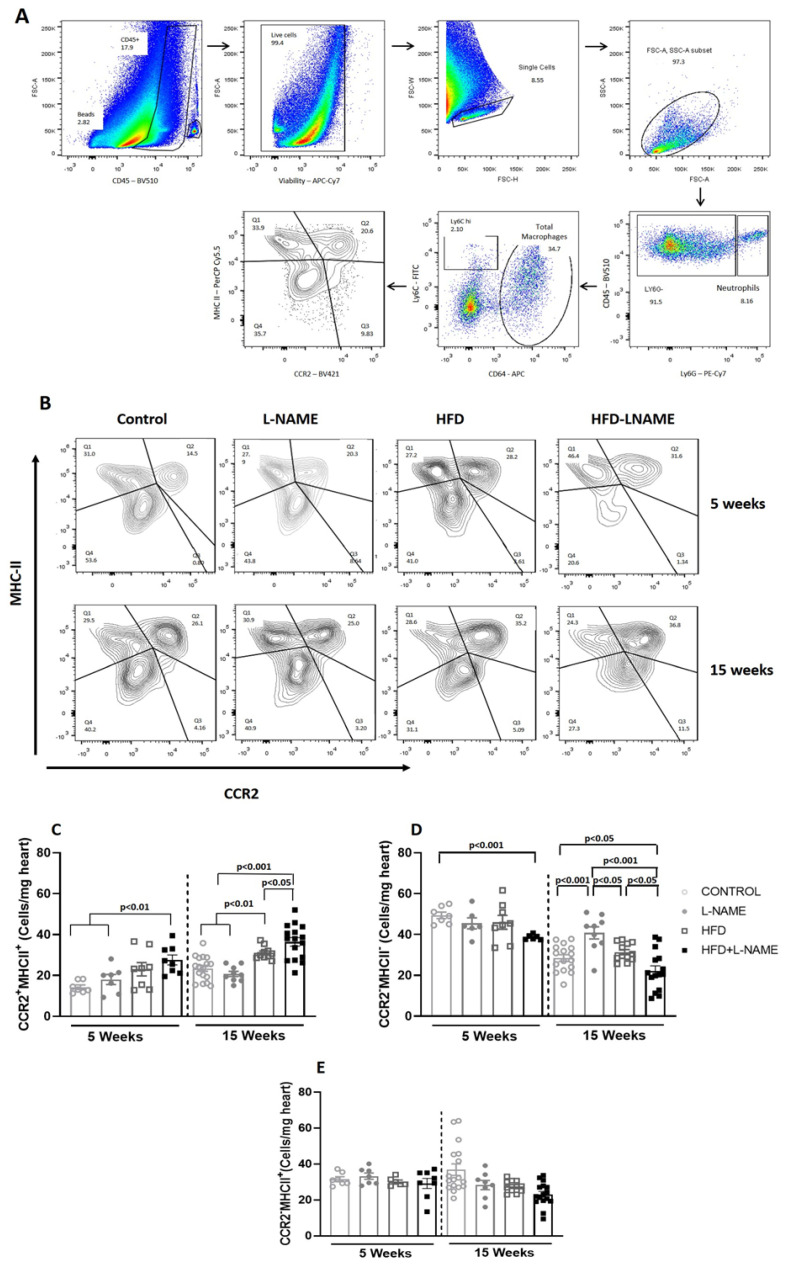
Cardiac macrophage subsets in mice after 5 and 15 weeks of dietary regimens. Groups as in Figure 1. For the determination of macrophage subtypes, the following antibodies were used: CD64-APC, -CCR2-BV421, MHCII-PerCP. The samples were acquired in FACS Canto II (BD) and analyzed with FlowJo v10.0.7 software. Results are presented as mean ± SEM. One-way analysis of variance (ANOVA) followed by Sidak’s multiple-comparisons test was performed. Numbers above square brackets show significant *p*-values. (**A**). Flow cytometry gating strategy for identification of cardiac macrophage subsets. (**B**) Representative gate plots of cardiac macrophages in each experimental group. (**C**) Quantitation of proinflammatory macrophages (CCR2^+^MHCII^+^) per milligram of heart tissue after 5 (F = 5.31; *p* < 0.01) and 15 weeks (F = 19.47; *p* < 0.0001). (**D**) Number of protective resident macrophages (CCR2^−^MHCII^−^) per milligram of heart tissue after 5 (F = 3.02; *p* < 0.05) and 15 weeks (F = 10.27; *p* < 0.001). (**E**) Number of macrophages maintained partially through local proliferation and also replaced by monocytes (CCR2^−^MHCII^+^) per milligram of heart tissue after 5 and 15 weeks.

Low-grade chronic systemic inflammation is implicated in progression from pre-HFpEF to symptomatic HFpEF [4]. One of the mechanisms proposed to explain the link between low-grade chronic systemic inflammation and increased diastolic LV stiffness in HFpEF is myocardial infiltration by activated macrophages. Figure 8A represents the flow cytometry gating strategy for detecting cardiac macrophage subtypes; Figure 8B shows representative gate plots of cardiac macrophages in each experimental group. At week 5, myocardial proinflammatory CCR2^+^MHCII^+^ macrophages increased only in HFD+L-NAME (pre-HFpEF) (93% increase vs. control; *p* < 0.001; Figure 8C). At week 15, CCR2^+^MHCII^+^ cells increased in HFD (39% increase; *p* < 0.001) and in HFD+L-NAME (60% increase; *p* < 0.001) vs. control (Figure 8C). From 5 to 15 weeks, proinflammatory macrophages increased significantly in HFD (37.6% increase; *p* < 0.05) and HFD+L-NAME (32% increase; *p* < 0.05).

In contrast, at week 5, resident reparative CCR2^−^MHCII^−^ macrophages decreased only in HFD+L-NAME (pre-HFpEF) vs. control (24% decrease; *p* < 0.001; Figure 8D). As HFpEF progressed, resident reparative macrophages decreased in HFD+L-NAME (30% decrease from week 5 to 15; *p* < 0.01). CCR2^−^MHCII^+^ resident macrophages (maintained partly by local proliferation and partly by monocytes) did not change at 5 or 15 weeks in the experimental groups.

**Figure 9:**
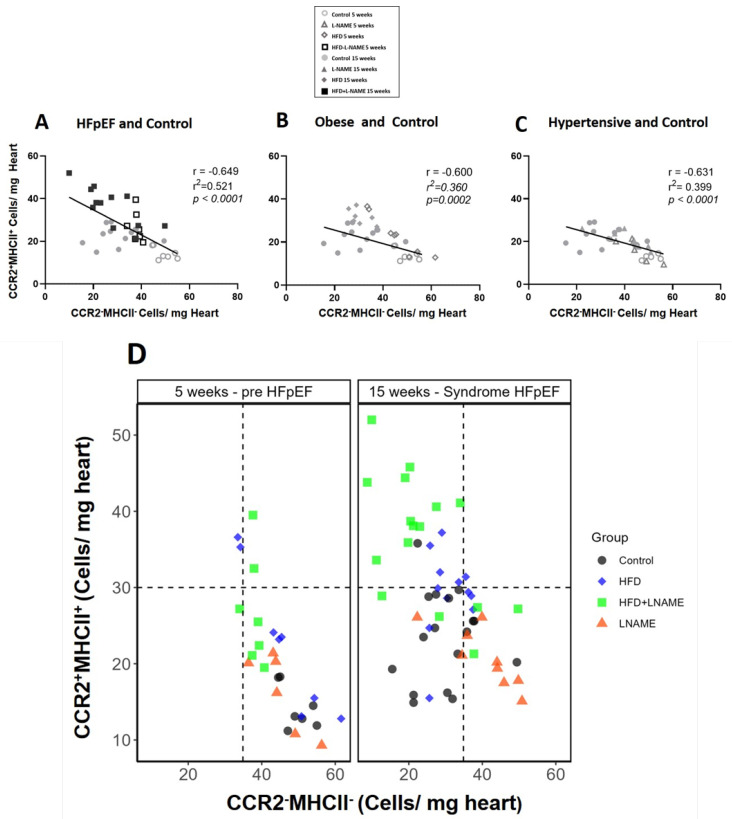
Relationships between proinflammatory CCR2^+^MHCII^+^ and protective CCR2^−^ MHCII^−^ cardiac macrophages after 5 and 15 weeks of dietary regimens. Correlation analyses for: (**A**) HFD+L-NAME and controls, *r* = −0.649, *r*^2^ = 0.521, *p* < 0.0001, *n* = 38; (**B**) HFD and controls, *r* = 0.600, *r*^2^ = 0.360, *p* < 0.001, *n* = 34; and (**C**) L-NAME and controls, *r* = −0.631, *r*^2^ = 0.399, *p ˂* 0.0001, *n* = 34. (**D**) Summary of cardiac macrophage subsets across all models at 5 weeks and 15 weeks.

An inverse relationship was found between CCR2^+^MHCII^+^ and CCR2^−^MHCII^−^ macrophages at 5 and 15 weeks in HFD+L-NAME (*r* = −0.649, *p* < 0.0001), HFD (*r* = −0.600, *p* = 0.001), and L-NAME (*r* = −0.631, *p* < 0.0001) (Figure 9A–C). In the progression of HFpEF, the proinflammatory macrophages increased and resident reparative macrophages significantly decreased (Figure 9D).

### 2.3. Circulating TNF-α and IL-10 During HFpEF Progression

**Figure 10:**
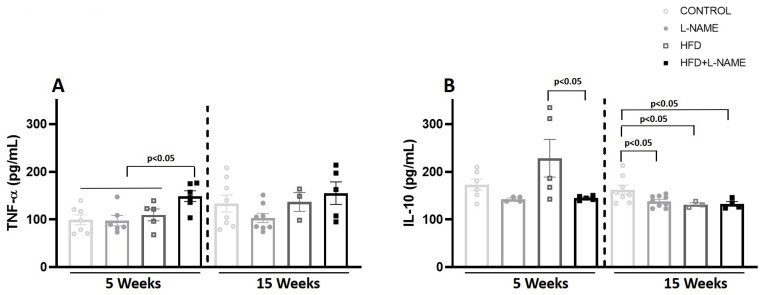
Plasma TNF-α and IL-10 at 5 and 15 weeks of dietary regimens. Groups as in Figure 1. (**A**) Plasma TNF-α (pg/mL) and (**B**) Plasma IL-10 (pg/mL) by ELISA measurements. Results are presented as mean ± s.e.m. One-way analysis of variance (ANOVA) followed by Sidak’s multiple-comparisons test was performed. Numbers above square brackets show significant *p-* values. (**A**) TNF-a plasma levels (pg/mL) at 5 (F = 4.67, *p* < 0.01) and 15 weeks (F = 1.67, ns). (**B**) IL-10 plasma levels (pg/mL)) at 5 (F = 3.45, *p* < 0.04) and 15 weeks (F = 3.79, *p* < 0.03).

At week 5, plasma TNF-α increased significantly in HFD+L-NAME (pre-HFpEF) vs. control (148.8 ± 11.2 vs. 99.1 ± 27.6 pg/mL; *p* < 0.01; Figure 10A). At 15 weeks, no between-group differences were observed. Plasma IL-10 increased significantly only in HFD at week 5 vs. other groups (Figure 10B). In contrast, at week 15 plasma IL-10 levels decreased significantly in L-NAME, HFD and HFD+L-NAME vs. control.

### 2.4. Cardiac AMP-Activated Protein Kinase (AMPK) Activation During HFpEF Progression

**Figure 11:**
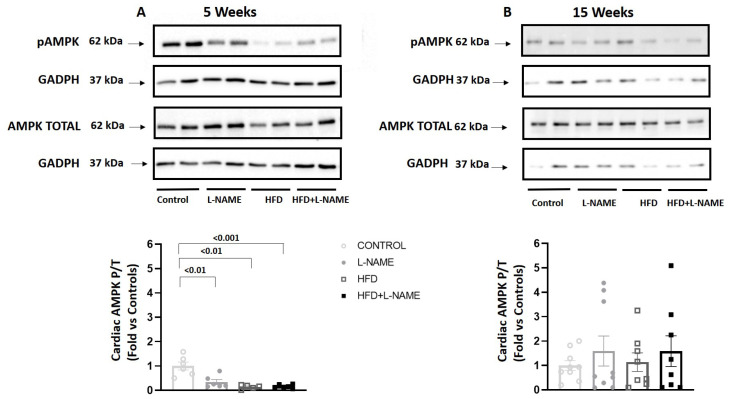
Cardiac AMPK activity in mice after 5 and 15 weeks of dietary regimens. Groups as in Figure 1. Cardiac AMPK activation assessed as *p*-AMPK(Thr172)/total AMPK by Western blot. AMPK activity at 5 (**A**) and 15 weeks (**B**) of experimental regimens. Results are presented as mean ± s.e.m. One-way analysis of variance (ANOVA) followed by Sidak’s multiple-comparisons test was performed. Numbers above square brackets show significant *p*-values. AMPK activity at 5 (F = 22.6, *p* < 0.001, **A**) and 15 weeks (F = 2.8, ns, **B**) after different dietary regimens.

AMPK activation, assessed as the ratio of phosphorylated to total AMPK by immunoblot (Figure 11), was significantly reduced at 5 weeks in the L-NAME (*p* = 0.008; F = 1.55), HFD (*p* = 0.003; F = 7.5), and HFD+L-NAME (*p* = 0.001; F = 9.6) groups vs. controls (Figure 11A). At 15 weeks, no significant between-group differences were detected (Figure 11B).

## 3. Discussion

A major finding in this model of heart failure with preserved ejection fraction (HFpEF) is that myocardial macrophage populations shift during disease progression. The early preclinical stage (week 5 of experimental treatment) was characterized by decreasing levels of resident reparative CCR2^−^MHCII^−^ macrophages in parallel with elevated proinflammatory CCR2^+^MHCII^+^ macrophage levels. These changes were associated with higher circulating TNF-α (consistent with low-grade inflammation), decreased myocardial AMP-Activated Protein Kinase (AMPK) activation, and increased myocardial fibrosis. At onset of symptomatic HFpEF (15 weeks), proinflammatory CCR2^+^MHCII^+^ macrophages remained elevated, whereas resident reparative CCR2^−^MHCII^−^ macrophages had normalized.

Two hallmarks of HFpEF remodeling are interstitial fibrosis and hypertrophy [11]. In this two-hit model, mice fed a high-fat diet (HFD) and L-NAME in drinking water for 15 weeks developed HFpEF compared with mice fed control chow, HFD alone, or L-NAME alone. HFpEF mice showed left atrial enlargement, left ventricular hypertrophy, diastolic dysfunction with normal systolic function, cardiomyocyte hypertrophy, myocardial fibrosis and decreased exercise capacity, consistent with other mouse models of the disease [10,12,13].

An ideal animal HFpEF model should manifest the key phenotypes observed in human HFpEF, and most two-hit models combine some forms of hypertensive and metabolic stress in different ways [14]. The few available experimental mouse models generally exhibit hypertension (HT) (via various mechanisms), obesity, and insulin resistance or type 2 diabetes mellitus [10,12,13], as in the model used here. These features characterize metabolic syndrome, which is highly prevalent worldwide, with reported rates ranging from 24.3% to 44.2% in parts of Asia, Europe, Mexico, and the United States [15,16]. To generate the present model, C57BL/6N mice were fed a HFD to induce metabolic stress (obesity and metabolic syndrome) and given L-NAME (an NOS inhibitor) in drinking water to induce mechanical stress via HT caused by NOS inhibition of nitric oxide synthesis [10]. To further confirm metabolic stress in our experimental model, this study explored the interrelation between HFpEF and blood lipid metabolism. TG, LDL, CHO significantly increased and HDL demonstrated a visible decrease. These alterations in blood lipids metabolism emphasize the definitive irregularities observed in HFpEF patients.

No previous studies have observed early changes in cardiac macrophage subpopulations in heart failure with preserved ejection fraction, which is associated with an early and transient increase in circulating pro-inflammatory TNF-α levels. This study found that changes in circulating TNF-α levels paralleled changes in cardiac macrophage subpopulations. Macrophage activation is a hallmark of chronic low-grade inflammation, which is involved in the pathogenesis of diabetic complications [17]. As in many non-lymphoid organs, macrophages are the predominant immune cell type in the heart [11]. Macrophages are a heterogeneous group of phagocytic immune cells that perform diverse functions in maintaining tissue homeostasis and responding to injury. They reside in the interstitial space in direct contact with cardiomyocytes, endothelial cells, and fibroblasts [4]. Inflammation has been proposed as an inciting event in HFpEF pathogenesis but a reactive event during HFrEF [11]. In the early stages of HF development (pre-HFpEF, 5 weeks of experimental regimen), we observed a decrease in resident reparative CCR2^−^MHCII^−^ macrophages and an increase in proinflammatory CCR2^+^MHCII^+^ macrophages in the HFD+L-NAME group. These changes were associated with early myocardial fibrosis and diastolic dysfunction, likely due to the contribution of high blood pressure, as similar findings were present in the L-NAME-only group at 5 weeks, in which HT was accompanied by increased proinflammatory CCR2^+^MHCII^+^ macrophages, myocardial fibrosis, and diastolic dysfunction.

In this study, a consistent inverse relationship between proinflammatory CCR2^+^MHCII^+^ and reparative CCR2^−^MHCII^-^ myocardial cells was observed not only in the HFpEF model but also in the hypertensive and obese mice, suggesting that these risk factors contribute to the pathogenesis of cardiac remodeling by altering immune-cell phenotypes in the heart, with implications for HFpEF prevention.

Our data show that macrophages recruited to the heart early in the progression of HFpEF (5 weeks) rapidly adopt a proinflammatory phenotype increasing CCR2^+^MHCII^+^ macrophages. In parallel, cardiac AMPK activation is decreased. AMPK has an important role in macrophage metabolism, function and polarization [18]. AMPK is a ubiquitous sensor of energy and nutritional status in eukaryotic cells. It plays a key role in regulating cellular energy homeostasis and multiple aspects of cell metabolism [19]. During macrophage polarisation, AMPK not only guides the metabolic programming of macrophages, but also counter-regulates the inflammatory function of macrophages and promotes their polarisation toward the anti-inflammatory phenotype [20]. It is known that AMPK activation reduces cardiac inflammation by inhibiting the nuclear factor-kappa (NF-kB) pathway, a key regulator of inflammation in macrophages. This leads to reduced production of pro-inflammatory cytokines (e.g., IL-1b and TNF-α), minimizing tissue damage in the heart [21]. Both resident and infiltrating macrophages in the heartexpress AMPK subunits (e.g., AMPKα1). The constitutive expression of AMPKα1 results in decreased production of pro-inflammatory cytokines and increased production of IL-10 in macrophages [22]. Studies show that AMPKα1 deletion in myeloid cells (macrophage lineage) worsens cardiac disfunction after myocardial infarction [23]

An early, transient reduction in myocardial AMPK phosphorylation was also observed in this two-hit HFpEF model, possibly induced by both HT and obesity, as it was likewise present in the L-NAME and HFD groups at 5 weeks. AMPK has been shown to inhibit cardiac hypertrophy [24]. Activated AMPK suppresses several anabolic pathways, including protein synthesis, via the *mTOR/p70S6K* and *eEF2* pathways, and promotes catabolic pathways such as glycolysis to maintain cellular energy balance [24]. In the current HFpEF model, the early reduction in AMPK phosphorylation was not associated with myocardial hypertrophy at 5 weeks. At 15 weeks, when LVH was evident in the HFD+L-NAME group, myocardial AMPK phosphorylation levels were similar to those in controls. A compensatory normalization of AMPK activation in the HFD+L-NAME group at 15 weeks cannot be excluded.

The normal heart is seeded with resident macrophages and embryonic progenitors derived from the yolk sac that are not replenished by circulating monocytes under stable conditions [7]. Monocyte-derived macrophages can be distinguished by expression of C-C chemokine receptor 2 (*CCR2*), which critically regulates their mobility and recruitment in response to C-C motif chemokine ligand 2 (CCL2; monocyte chemoattractant protein), also known as monocyte chemoattractant protein [25].

In humans, a prospective study used endomyocardial biopsies to assess monocyte/macrophage infiltration by CD68 expression in patients with HFpEF, HFrEF, and controls. Among HFpEF patients, 93% exhibited myocardial fibrosis and 88% displayed myocyte hypertrophy [26]. CD68^+^ macrophage infiltration was greater in HFpEF than in controls or HFrEF [27]. These results indicate that fibrosis, hypertrophy, and inflammation due to macrophage infiltration are highly prevalent in HFpEF myocardial remodeling. In contrast, myocardial macrophage density did not differ between patients with HT vs. age- and sex-matched controls [27].

To date, there is no direct clinical evidence demonstrating a causal role for macrophages in HFpEF progression. Glezeva et al. analyzed peripheral blood monocyte phenotypes and plasma markers of monocyte activation in three groups: (1) asymptomatic HT with normal echocardiogram; (2) asymptomatic LV diastolic dysfunction; and (3) HFpEF with previous hospitalization for HF. Classical proinflammatory cytokines (TNF-α, IL-12, IL-6, MCP-1, and CCL10) and anti-inflammatory markers (CCL17, CCL18, and soluble CD163) were elevated in LV diastolic dysfunction and HFpEF. Classical proinflammatory monocytes were 19% and 21% higher in LV diastolic dysfunction and HFpEF, respectively, and anti-inflammatory monocytes were 21% higher in HFpEF [28]. Conversely, levels of the anti-inflammatory CD163 (M2) macrophage receptor were lower in HFpEF, consistent with an enhanced inflammatory response [28]. The authors concluded that peripheral inflammation and monocytic differentiation into anti-inflammatory/profibrotic M2 macrophages are likely associated with HFpEF and its preceding phase of asymptomatic LV diastolic dysfunction [28]. From a clinical perspective, it could be possible that by measuring a proinflammatory cytokine such as blood TNF-α and by assessing monocytic differentiation into M2 macrophages in peripheral mononuclear cells, a precise evolving cardiac remodelling picture of human HFpEF could be obtained.

The present study is subject to certain limitations. First, establishing a causal role of CCR2^+^MHCII^+^ macrophages in cardiac remodeling and HFpEF progression remains unproven. They would require selective depletion or blockade of their infiltration (e.g., with a receptor antagonist or antibody). Second, despite HFpEF being particularly prevalent in women, the results presented here are from male mice only. Further comparative studies in female animals are necessary to determine whether different responses occur in both genders in this experimental model. Besides, the issue of extramedullary hematopoiesis in the spleen, involving the production and mobilization of immune cells to the heart and the production of cytokines [29], alongside cardiac remodeling, was not assessed here. Nonetheless, there is a possibility that this could be occurring in this HFpEF model.

## 4. Materials and Methods

### 4.1. Experimental Model

All procedures complied with the Guide for the Care and Use of Laboratory Animals published by the U.S. National Institutes of Health (8th edition, 2011 update) and all relevant ethical regulations and were approved by the Institutional Animal Care and Use Committee of the Pontificia Universidad Católica de Chile (ID 210614003). Adult male C57BL/6N mice (12 weeks old) were subjected to two-hit stress: metabolic (obesity and metabolic syndrome) and mechanical (hypertension induced by constitutive NO synthase suppression), as described previously [10]. Mice were randomized to one of four groups, standard chow (control, *n* = 8), L-NAME (0.65 g L^−1^ in drinking water, *n* = 8), high-fat diet (HFD; 60% kcal from fat, *n* = 12), or HFD+L-NAME (two-hit model, *n* = 12), for 5 or 15 weeks.

### 4.2. Tail-Cuff Blood Pressure

Systolic and diastolic blood pressures were measured noninvasively in conscious mice using the tail-cuff method (CODA system, Kent Scientific, Torrington, CT, USA). Mice were placed in individual holders on a temperature-controlled platform (37 °C), and recordings were performed under steady-state conditions. Before testing, mice were trained to tolerate short-term restraint. Blood pressure was recorded for at least four consecutive days, and readings were averaged from at least 20 measurements per session.

### 4.3. Exercise Exhaustion Test

After three days of treadmill acclimatization, an exhaustion test was performed as described [10]. Mice ran uphill (10°) on a treadmill (Harvard Apparatus, Holliston, MA, USA), starting with a 5 min warm-up at 5 m·min^−1^, followed by 14 m·min^−1^ for 2 min. Thereafter, speed increased by 2 m·min^−1^ every 2 min until exhaustion, defined as failure to resume running within 10 s of contact with the stimulus grid. Running time and distance were recorded.

### 4.4. Intraperitoneal Glucose Tolerance Test

Intraperitoneal glucose tolerance tests (ipGTT) were performed by glucose injection (2 g·kg^−1^ in saline) after a 6 h fast. Tail blood glucose (mg·dL^−1^) was measured with a glucometer at 0 (baseline) and 15, 30, 45, 60, and 120 min after glucose administration [10].

### 4.5. Blood Lipid

After 5 and 15 weeks, blood samples were collected and placed in heparin anticoagulant tubes for 2 h. After that, the samples were separated at 3000 rpm/heart for 15 min at 2–8 °C. The triglyceride, total cholesterol, HDL cholesterol, and LDL cholesterol levels were determined by a biochemical analyzer.

### 4.6. Conventional Echocardiography and Doppler Imaging

Transthoracic echocardiography was performed using a Vivid i GE system with an i12L-RS (4.5–11.5 MHz) transducer. LVEF and other systolic indices were obtained from short-axis M-mode scans at the midventricular level (papillary muscles visible) in conscious, gently restrained mice [30]. Apical four-chamber views were acquired in anesthetized mice for diastolic measurements using pulsed-wave and tissue Doppler at the mitral valve. Anesthesia was induced with 4% isoflurane and confirmed by lack of response to firm pressure on a hind paw. During echocardiogram acquisition, isoflurane was reduced to 1.0–1.5% under temperature-controlled conditions, adjusted to maintain heart rate at 415–460 bpm. Recorded parameters included heart rate; LV end-diastolic diameter (LVDD); LV end-systolic diameter (LVSD); end-diastolic septal wall thickness (SWT); LV end-diastolic posterior wall thickness (LVPW); LV fractional shortening (LVFS); and LVEF. LV mass was calculated by the area–length method [31] using LV mass (g) = 1.053 × ([LVDD + SWT + LVPW]^3^ − [LVDD]^3^). Diastolic dysfunction was assessed by E/A and E/e′ ratios from 2D parasternal short-axis views in diastole.

### 4.7. Left Ventricular Hypertrophy Measurement

LV hypertrophy was calculated as LV mass (g) normalized to tibial length (TL, cm). Morphological and morphometric analyses were performed by light microscopy of paraffin-embedded mid-ventricular transverse sections (5 µm) stained with hematoxylin and eosin [12]. Cardiomyocyte size (area and perimeter) was determined as described [32].

### 4.8. Cardiac Fibrosis

LV fibrosis was assessed morphometrically. At 5 or 15 weeks, mice were euthanized under deep anesthesia. Hearts were rinsed in saline, weighed, fixed in 4% formalin in PBS for 12 h, and embedded in paraffin. Transverse sections (5 µm) were stained with Picrosirius Red to quantify fibrosis [33].

### 4.9. Flow Cytometry

Single-cell suspensions were generated from saline-perfused hearts by mincing and digesting in Dulbecco’s modified Eagle Medium (DMEM) with collagenase I (450 U·mL^−1^), hyaluronidase (60 U·mL^−1^), and DNase I (60 U·mL^−1^) for 1 h at 37 °C. Enzymes were quenched with Hank’s balanced salt solution (HBSS) supplemented with 2% fetal bovine serum (FBS) and 0.2% BSA, and suspensions were filtered through 40 µm strainers. Red blood cells were lysed with ammonium-chloride–potassium (ACK) buffer for 5 min on ice. Samples were washed with DMEM and centrifuged at 300× *g* for 5 min; supernatants were discarded and cells resuspended in 1 mL phosphatase buffered saline (PBS) for counting [34]. Viability was assessed with Zombie-APC-Cy7 (Biolegend, San Diego, CA, USA; #423107, 15 min, 4 °C, dark). Immune cells were gated as CD45^+^. Neutrophils: Ly6G^+^CD64^−^. Monocytes: Ly6G^−^. Macrophages: Ly6G^−^CD64^+^. Macrophage subtypes: CCR2^−^MHCII^−^, CCR2^−^MHCII^+^, CCR2^+^MHCII^−^, CCR2^+^MHCII^+^. The antibody panel consisted of the following antibodies: CD45-PerCP-Cy5.5 (clone 30-F11, Biolegend #103138), Ly6G-PE-Cy7 (clone 1A8, Biolegend #127618), Ly6C-FITC (clone HK1.4, Biolegend #128006), CD64-APC (clone X54-5/7.1, Biolegend #139306), MHCII-PerCP (clone M5/114.15.2, Biolegend # 107624), CCR2-BV421 (clone SA203G11, Biolegend1500605) [35]. Flow cytometry was performed on a BD FACSCanto II and analyzed in FlowJo.

### 4.10. Plasma Insulin, IL-10 and TNF-α

Plasma was obtained by centrifugation (1600*g*, 15 min, 4 °C). Plasma insulin, interleukin-10 (IL-10) and tumor necrosis factor-α (TNF-α) were measured by ELISA (Millipore and CUSABIO). Briefly, 50 µL of standard or serum was added to microplate wells precoated with monoclonal antibody for insulin, IL-10 or TNF-α and incubated for 2 h at room temperature. After rinsing off any unbound substances, an enzyme-linked polyclonal antibody against insulin, IL-10 or TNF-α was added and incubated for 2 h. After washing, 100 µL substrate solution was added for 30 min, followed by 100 µL stop solution for color development. Optical density was read at 450 nm using a microplate reader. Insulin, IL-10 and TNF-α assays had a minimum sensitivity of: 0.1 ng/mL (insulin), 0.78 pg/mL (IL-10) and 1.56 pg/mL (TNF-α).

### 4.11. Western Blot Analysis to Determine Cardiac Adenosine Monophosphate (AMP)–Activated Protein Kinase (AMPK) Activity

AMPK activity was assessed as the ratio of phosphorylated to total AMPK. Soluble protein fractions were heated for 5 min at 95 °C in SDS sample buffer (375 mM Tris–HCl pH 6.8, 6% SDS, 48% glycerol, 9% 2-mercaptoethanol, 0.03% bromophenol blue). Equal protein amounts were run on 5% stacking/8% resolving SDS-PAGE (80 V) and transferred to nitrocellulose (450 mA, 1 h, on ice). Membranes were blocked with 5% milk (1 h, room temperature) and incubated overnight (4 °C) with primary antibodies. Signals were detected by chemiluminescence (ECL plus kit, Perkin Elmer, MA, USA, which contains the substrate for horseradish peroxidase, HRP). Digital images were captured on a Syngene G-Box and quantified by densitometry (UN-SCAN-IT™ v6.1, Silk Scientific Inc., Orem, UT, USA).

Blots were incubated overnight with anti-*p*-AMPK (phosphor-AMPK rabbit mAb 1:2000, 2535) and anti-AMPKα (rabbit polyclonal, 1:1000, Cell Signaling 2532). Blots were then washed and incubated with an HRP-conjugated goat anti-rabbit IgG secondary antibody (1:7500, 2 h; Thermo Scientific, Rockford, IL, USA, Cat. 31460). Protein loading was confirmed by Ponceau S staining [36].

### 4.12. Statistical Analyses

At 5 weeks, each experimental group contained controls, *n* = 8; L-NAME, *n* = 8; HFD, *n* = 12; and HFD+L-NAME, *n* = 12. At 15 weeks, the same experimental groups contained *n* = 12. Data are mean ± SEM. Comparisons used one-way ANOVA with Sidak’s multiple-comparisons test (SPSS 21.0). *p* < 0.05 was considered statistically significant. Graphs were created in GraphPad Prism 5.

## 5. Conclusions

This two-hit model (high-fat diet and L-NAME) induced heart failure with preserved ejection fraction (HFpEF), accompanied by obesity, glucose intolerance, and hypertension. Myocardial tissues exhibited increased proinflammatory CCR2^+^MHCII^+^ macrophage levels, beginning in preclinical stages and persisting through HFpEF onset. An initial decrease in resident reparative CCR2^−^MHCII^−^ macrophages was also observed, with levels normalizing by HFpEF onset. The early shifts in macrophage populations were associated with low-grade inflammation and myocardial remodeling preceding the onset of HFpEF.

## Figures and Tables

**Table 1:** Blood lipids levels of mice after 5 and 15 weeks feeding with control chow, L-NAME, HFD or HFD+L-NAME. Mean ± SEM. Symbols: *** *p* < 0.001, ** *p* < 0.01. * *p* < 0.05 vs. control chow and L-NAME at 5 and 15 weeks. Post hoc multiple comparisons after a significant ANOVA were performed with Sidak’s multiple-comparisons test.

	5 Weeks						15 Weeks					
Parameter	CONTROL	L-NAME	HFD	HFD+ L-NAME	F Value	*p*-Value	CONTROL	L-NAME	HFD	HFD+ L-NAME	F Value	*p*-Value
*n*	8	8	12	12			8	8	12	12		
TG, mmol/L	2.81 ± 0.31	2.52 ± 0.27	3.54 ± 0.18 ***	3.50 ± 0.21 ***	10.9	0.0001	2.92 ± 0.29	2.44 ± 0.17	4.02 ± 0.23 ***	3.87 ± 0.20 ***	10.13	0.0001
LDL, mmol/L	1.42 ± 0.14	1.52 ± 0.19	3.39 ± 0.23 ***	3.19 ± 0.18 ***	12.93	0.0001	1.52 ± 0.18	1.23 ± 0.15	3.87 ± 0.11 ***	3.69 ± 0.20 ***	13.67	0.0001
HDL, mmol/L	2.57 ± 0.23	2.82 ± 0.11	2.29 ± 0.18 *	2.20 ± 0.21 *	4.12	0.03	2.71 ± 0.22	2.78 ± 0.15	2.12 ± 0.13 **	2.10 ± 0.22 **	2.73	0.001
CHO, mmol/L	2.14 ± 0.15	2.21 ± 0.22	3.85 ± 0.21 ***	3.65 ± 0.28 ***	8.92	0.0001	2.23 ± 0.18	2.34 ± 0.25	4.31 ± 0.19 ***	3.78 ± 0.27 ***	6.63	0.0001

**Table 2:** Short- (5 weeks) and long-term (15 weeks) effects of obesity and hypertension on LV function and morphometry. Mean ± SEM. SWT, septal wall thickness; LVPWT, LV posterior wall thickness; HR, heart rate; LVEDD, LV end-diastolic diameter; LVESD, LV end-systolic diameter; LVEF, LV ejection fraction; LVFS, LV fractional shortening; LV mass from echo formula in Methods; TL, tibial length; LA, left atrium; A, late diastolic mitral inflow velocity; E, early diastolic mitral inflow velocity; e′, early diastolic mitral annular tissue velocity; NS, non significant. Symbols: *** *p* < 0.01 vs. all groups (5 and 15 weeks); ^††^ *p* < 0.01 vs. L-NAME (5 weeks); ^♢♢♢^ *p* < 0.001 vs. control and HFD (5 and 15 weeks); ^‡‡‡^ *p* < 0.01 vs. control, HFD, and L-NAME (5 weeks). Post hoc multiple comparisons after a significant ANOVA were performed with Sidak’s multiple-comparisons test.

	5 Weeks						15 Weeks					
Parameter	CONTROL	L-NAME	HFD	HFD+ L-NAME	F Value	*p*-Value	CONTROL	L-NAME	HFD	HFD+ L-NAME	F Value	*p*-Value
*n*	8	8	12	12			8	8	12	12		
LVSWT, mm	0.69 ± 0.02	0.66 ± 0.00	0.69 ± 0.02	0.68 ± 0.01	0.90	NS	0.68 ± 0.01	0.68 ± 0.01	0.76 ± 0.01 ***	0.74 ± 0.01 ***	12.12	0.000
LVPWT, mm	0.68 ± 0.01	0.67 ± 0.02	0.66 ± 0.00	0.69 ± 0.01	0.79	NS	0.68 ± 0.01	0.67 ± 0.01	0.74 ± 0.01 ***	0.74 ± 0.01 ***	13.67	0.000
HR, bpm	232 ± 4	225 ± 7	226 ± 4	226 ± 5	0.31	NS	233 ± 2	224 ± 1	226 ± 34	233 ± 1	2.73	NS
LVEDD, mm	2.86 ± 0.05	2.87 ± 0.03	2.92 ± 0.03	2.86 ± 0.04	0.64	NS	2.94 ± 0.01	2.93 ± 0.02	3.06 ± 0.03 ***	2.97 ± 0.01	6.63	0.001
LVESD, mm	1.85 ± 0.06	1.83 ± 0.05	1.95 ± 0.02	1.90 ± 0.03	2.01	NS	1.95 ± 0.02	1.98 ± 0.02	2.04 ± 0.04 ^††^	1.98 ± 0.06	4.41	0.04
LVEF, %	72.75 ± 2.03	72.00 ± 1.43	69.58 ± 1.14	70.00 ± 1.06	1.10	NS	69.88 ± 0.83	68.22 ± 0.1	70.47 ± 1.07	70.70 ± 1.08	1.30	NS
LVFS, %	35.37 ± 1.75	35.50 ± 1.13	33.66 ± 0.93	34.28 ± 0.72	0.64	NS	34.61 ± 1.01	32.72 ± 0.69	34.11 ± 0.76	33.35 ± 1.11	0.80	NS
LV mass, g	58.94 ± 1.14	53.39 ± 1.33	54.74 ± 1.86	54.53 ± 1.69	0.61	NS	57.52 ± 1.23	55.55 ± 1.13	68.43 ± 1.70 ***	64.66 ± 1.07 ***	9.24	0.000
LV mass/TL, g/cm	34.11 ± 1.12	31.50 ± 0.86	30.47 ± 0.73	31.80 ± 1.10	1.26	NS	32.96 ± 0.82	31.95± 0.79	38.15 ± 0.78 ***	37.24 ± 0.66 ***	10.15	0.000
LA, mm	1.57 ± 0.03	1.57 ± 0.03	1.53 ± 0.03	1.61 ± 0.04	1.15	NS	1.56 ± 0.03	1.62 ± 0.02	1.81 ± 0.02 ***	1.84 ± 0.02 ***	31.53	0.000
E/e’	20.37 ± 0.75	32.68 ± 1.92 **^♢♢♢^**	21.50 ± 0.97	39.45 ± 1.72 ^‡‡‡^	19.25	0.000	24.12 ± 1.91	38.56 ± 2.76 **^♢♢♢^**	24.89 ± 2.69	43.53± 1.97 ***	30.34	0.000
E/A	1.31 ± 0.54	1.82± 0.53 **^♢♢♢^**	1.26 ± 0.09	1.96 ± 0.32 ^‡‡‡^	15.89	0.000	1.22 ± 0.65	1.94 ± 0.83 **^♢♢♢^**	1.37 ± 0.69	2.35 ± 1.18 ***	14.87	0.000

## Data Availability

The datasets used and/or analyzed during the current study are available from the corresponding authors on reasonable request.
